# A Place Where Diplomas Are Not Needed

**Published:** 1881-04

**Authors:** 


A Place where Diplomas are Not Needed.—"The Maine Reports," says the *Boston Advertiser*, "record a decision which overruled the decision of a lower court, to the effect that a clairvoyant, who expressly disclaims any medical or surgical knowledge, was entitled to recover for 'medical services,' because she had visited and examined the sick person and prescribed.

"There is no question," continues the journal referred to, "that the statute was fairly represented in the opinion of the court, but what must be thought of a law which opens wide the door to the operations of any who think they can make a living more easily by dealing with disease than by going out to service, or by driving a truck or a peddlar's cart? If so be morality is certified to, it matters not that the man or woman cannot read, write, count, see, hear or be able to do any of the things that a doctor ought to do. The most impudent imposter, his certificate secured, may put out his sign and practice upon the fear or shame of those weak enough to patronize him, charge heavily for his undesirable services, and the law says, pay in full, or he may make you discharge the debt to him and the court costs besides. If any quack finds the atmosphere of another State oppressive, if the statutes fail to shield him at the expense of those who are his victims, let him take courage. Main offers him a shelter. Her statute provides for his well-being. He need bring no knowledge, no outfit, no pill or potion. If there be with him the certificate required, the law ranks him with the wise and noble of the profession which deserves the high repute it has attained.—*Medical Record.*

